# Studying the Diabetic Foot at Risk Using a 60-Second Foot Screening Tool and the Importance of the Categories of the Foot at Risk in Diabetes Patients at a Tertiary Care Center in East India

**DOI:** 10.7759/cureus.72615

**Published:** 2024-10-29

**Authors:** Kishore Kumar Behera, Uttam Kumar Soren, Binod Kumar Behera, Sujata Devi

**Affiliations:** 1 Endocrinology and Metabolism, All India Institute of Medical Sciences, Bhubaneswar, Bhubaneswar, IND; 2 General Medicine, Institute of Medical Sciences & SUM Hospital, Siksha 'O' Anusandhan University, Bhubaneswar, IND; 3 Community and Family Medicine, All India Institute of Medical Sciences, Bhubaneswar, Bhubaneswar, IND; 4 General Medicine, All India Institute of Medical Sciences, Bhubaneswar, Bhubaneswar, IND

**Keywords:** 10 gm monofilaments, diabetes mellitus, diabetic foot ulcer, diabetic peripheral neuropathy (dpn), foot amputation, foot at risk, foot care practice, loss of protective sensation (lops), peripheral arterial diseases, simplified 60-second diabetic foot screen tool (ssdfst)

## Abstract

Introduction

The etiology of a diabetic foot ulcer (DFU) is multifactorial. The three main components that are implicated in DFUs are foot deformity, repeated minor trauma to the foot, and diabetic peripheral neuropathy.

Aim and objectives

The study aims to find the prevalence of diabetes patients having a foot at risk using the Simplified 60-Second Diabetic Foot Screen tool (SSDFST). The objective is to ascertain the dispersal of various categories of the foot at risk in patients with diabetes and to find out the association of neuropathy with the various risk factors for the evolution of DFUs*.*

Materials and methods

This was a cross-sectional study comprising 128 patients; a detailed history and examination including neurological and vascular assessment were performed attending a tertiary care hospital. Patients were screened for the risk of diabetic foot using the SSDFST. The detection of loss of protective sensation (LOPS) using a simple 10-g monofilament test (10g M) was highly predictive of subsequent ulceration, which had been reported by the Seattle Diabetic Foot Study. The foot at risk was correlated with demographic and clinical features. Data were analyzed using descriptive and inferential statistics, significant at p = 0.05.

Results

Out of 128 patients, 92 (72%) and 36 (28%) were male and female, respectively. The mean duration of diabetes was 7.42 ± 6.23 years (range 1-27). The mean age and BMI of the study population were 53.13 ± 10.99 years and 25.93 ± 4.46 kg/m^2^, respectively. Out of 128 patients, 82 (64%) were normal without any risk factor for diabetic foot, and 46 (36%) patients had at least one risk factor for diabetic foot using the SSDFST. About 36% of patients were combinedly qualified for the foot at risk into (categories 1, 2, and 3), among which six (5%) were placed under category 1, 18 (14%) patients were classified under category 2 with LOPS + PAD, and 22 (17%) were placed under category 3 with a history of ulcer and/or amputation. The duration of diabetes, previous foot ulcer, deformity, absent pedal pulses, active ulcers, and neuropathy (p = 0.05) were significantly associated with neuropathy measured by 10g M.

Conclusions

Our study revealed that one-third of our patients had at least one risk factor for the diabetic foot using the SSDFST. About one-fifth of our patients had neuropathy detected by monofilaments. Meanwhile, two-fifth of the study population were aware of proper foot care practices.

## Introduction

The etiology of a diabetic foot ulcer (DFU) is multifaceted as described by Amin and Doupis [1]. The three principal components that ascertain the likelihood of ulceration in a diabetic foot are peripheral neuropathy, repeated minor trauma, and deformity [2]. The probability of getting a foot ulcer in a person in their lifetime is 15-25%, and the recurrence rate of subsequent ulcers is 50-70% for five years [3]. Diabetic patients with concurrent ulcers in the lower limbs are associated with developing amputation in about 15% [3]. If there is the presence of more than one of four features, such as a history of foot ulcers, bilateral peripheral diabetic neuropathy, lower limb peripheral vascular disease, and diabetic neuropathy, the incidence of getting a foot ulcer increases to 30% [4]. DFU can be preventable with proper foot care, and the Simplified 60-Second Diabetic Foot Screen tool (SSDFST) developed by Sibbald et al. [3] is useful for detecting a foot at risk for the development of foot ulcers. This tool comprises the following components: previous ulcers, history of amputation, deformity of feet, pedal pulse palpation, active ulcer, ingrown toenail, foot calluses, foot blisters and fissures, and use of monofilament testing for neuropathy [3]. This study aimed to identify various DFU risk factors among people living with diabetes (PLWD) in outpatient diabetic care settings using the novel SSDFST.

This work was presented as an e-poster at the European Congress of Endocrinology 2022 [5].

## Materials and methods

Study design and methodology

In a study by Sibbald RG et al. [3], 45% of the DM patients with risk factors will develop a foot ulcer in the future, and with the power of the study at 80%, significance level at 5%, and attrition rate at 20%, the calculated sample size was 125. This prospective cross-sectional observational study included all the DM patients attending the endocrinology department of All India Institute of Medical Sciences (AIIMS), Bhubaneswar, between December 2019 and December 2020. The study population was diagnosed with diabetes mellitus according to the American Diabetes Association (ADA) criteria [6]. A detailed history with a neurological and vascular foot assessment was performed. The institutional ethics committee of the institution approved the study (reference no. T/IM-NF/ Endoci /19/20).

Statistical analysis

Data analysis was done using IBM SPSS Statistics for Windows, Version 22.0 (released 2012, IBM Corp., Armonk, NY). Descriptive statistics were used to find the number, percentage, mean, and standard deviation depicted in the tables. The data were further analyzed using the chi-square test to examine relationships between risk levels for categorical variables. Statistical significance was set at p < 0.05.

Inclusion and exclusion criteria

Patients with T2DM diagnosed according to the ADA criteria [6] with either sex aged more than 18 years and <65 years were taken for study. Excluded from the study were patients with diabetes with severe cardiovascular diseases, liver diseases, renal diseases, drug intake (hormones, antiepileptic, anti-tubercular treatment (ATT), and glucocorticoids), and not willing to give consent for participation.

The 60-second foot screening tool

The simplified 60-second DFST developed by Sibbald RG et al. [3] can determine the number of components of the diabetic foot at risk including the previous history of foot ulcers and amputations, physical examinations (pedal pulses, musculoskeletal problems, etc.), and various foot lesions. It also registers the 10 g Semmes-Weinstein’s monofilament (SWM) examination for peripheral neuropathy [7]. Answers were noted as "yes" or "no." Solely a single "yes" indicates a risk for foot ulceration, infections, and/or amputation, and the patients should be sent to a podiatric clinic. If all answers are "no," then the patient should be asked for reappraisal in one year [7]. The screening test detects the high-risk diabetic foot. 

Risk categorization established on the comprehensive foot assessment and guideline/follow-up

The patient examination included a detailed dermatologic, musculoskeletal, and neurological examination with 10 g SWM (to perceive pressure sensation at the appropriate site) at the first, third, and fifth metatarsal heads and plantar surface of distal hallux to detect peripheral neuropathy. The efficacy in detecting loss of protective sensation (LOPS) using a simple 10 g monofilament test was highly anticipative of the later course of an ulceration that had been reported by the Seattle Diabetic Foot Study [8]. The patients were risk classified as per the protocol outlined based on Comprehensive Foot Examination and Risk Assessment into categories 0-3 [9]. The various risk categories of the diabetic foot and their link with previous foot care education, level of educational qualification, socioeconomic status, degree of medical care, and micro- and macrovascular complications were studied. 

## Results

Demographic characteristics of the study population

We recruited 128 diabetic subjects; 92 were male (72%) and 36 were female (28%). The mean duration of diabetes was 7.42 ± 6.23 years (range 1-27). Table 1 shows the demographic profile of the patients. The mean age of our patients was 53.12 ± 10.99 years (range 29-74). The mean BMI was 25.93 ± 4.46 kg/m^2^. Among the study population, about 21% were of normal weight (BMI <22.9 kg/m^2^), 20% were overweight (BMI of 23-24.9 kg/m^2^), and 59% were obese (BMI of >25 kg/m^2^), based on the Indian Asian BMI reference.

**Table 1:** Demographic parameters of the study population DM: diabetes mellitus; MCR shoes: microcellular rubber shoes

Variables	Number (N = 128)
Duration of DM (years)	
<5	55 (42.96%)
5-10	41 (32.03%)
>10	32 (25%)
Level of education	
Uneducated	13 (10.16%)
Primary (till the 5th class)	24 (18.75%)
Secondary (till the 10th class)	49 (38.28%)
Graduate and above	42 (32.81%)
History of previous foot care advice by a healthcare professional	
Present	36 (28.13%)
Absent	92 (71.88%)
Monthly income	
Up to 10,000/month	90 (70.31%)
10,001-20,000/month	15 (11.71%)
20,001-30,000/month	10 (7.81%)
>30,001/month	13 (10.16%)
Level of care	
Primary	51 (39.84%)
Secondary	34 (26.56%)
Tertiary	43 (33.59%)
Type of footwear worn by patients	
MCR Shoes	24 (18.75%)

Foot at risk screening for diabetic foot ulcers using the 60-second tool (2012) by Sibbald RG et al.

All the patients' feet were screened to look for the foot at risk using the SSDFST by Sibbald RG et al. [3]. The tool detected 10 (8%) with a previous ulcer, one (0.78%) with lower limb amputation, and 11 (9%) with an active ulcer. Loss of protective sensation (LOPS) was detected in 24 (19%) by a 10 g SWM test, and peripheral arterial disease (PAD) was present in 18 (14%) patients with the absence of peripheral pulsation by palpation. Other risk factors like deformity noted in five (4%) patients, ingrown toenails in 40 (31%), callus in 22 (17%), fissures in 27 (21%), and blisters in three (2%) subjects, as depicted in Table 2.

**Table 2:** Result of the 60-second screening tool (2012) for diabetic foot (n = 128)

Item	Yes (%)	No (%)
Previous ulcer	10 (7.81%)	118 (92.18%)
Previous amputation	01 (0.78%)	127 (99.21%)
Deformity	05 (3.90%)	123 (96.09%)
Absent pedal pulses	18 (14.06%)	110 (85.93%)
Active ulcer	11 (8.59%)	117 (91.40%)
Ingrown toenail	40 (31.25%)	88 (68.75%)
Calluses	22 (17.18%)	106 (82.81%)
Blisters	03 (2.34%)	125 (97.65%)
Fissures	27 (21.09%)	101 (78.90%)
Monofilament exam (neuropathy)	24 (18.75%)	104 (81.25%)

Out of 128 patients, 82 (64%) were normal without any risk factor for diabetic foot, and 46 (36%) patients had at least one risk factor for diabetic foot. The association of neuropathy was studied with various parameters like age, sex, BMI, HbA1c, duration of diabetes, education, per capita income, history of foot care advice, smoking, alcohol, tobacco, type of footwear used, and level of patient care received during their course of diabetes treatment. Our study revealed a positive association of neuropathy with the active ulcer, amputation, previous ulcer, ingrown toenails, blisters, calluses, deformity, duration of diabetes, and tobacco use with a p-value <0.05 (Table 3).

**Table 3:** Association of variables with monofilament testing DM: diabetes mellitus

Variables	Monofilament (+), n (%)	Monofilament (-), n (%)	P-value
Duration of DM			
0-5 years	6 (10.9%)	49 (89.1%)	0.024
5-10 years	7 (17.1%)	34 (82.9%)	
>10 years	11 (34.4%)	21 (65.6%)	
Tobacco			
Yes	9 (32.1%)	15 (15.0%)	0.040
No	19 (67.9%)	85 (85.0%)	
Previous ulcer			
Yes	10 (100%)	14 (11.9%)	0.001
No	0 (0.0%)	104 (88.1%)	
Previous amputation			
Yes	1 (100%)	23 (18.1%)	0.037
No	0 (0.0%)	104 (81.9%)	
Deformity			
Yes	4 (80.0%)	20 (16.3%)	0.001
No	1 (20.0%)	103 (83.7%)	
Absent pedal pulses			
Yes	6 (33.3%)	18 (16.4%)	0 .087
No	12 (66.7%)	92 (83.6%)	
Active ulcer			
Yes	11 (100.0%)	13 (11.1%)	0.001
No	0 (0.0%)	104 (88.9%)	
Ingrown toenail			
Yes	14 (35.0%)	10 (11.4%)	0.001
No	26 (65.0%)	78 (88.6%)	
Calluses			
Yes	8 (36.4%)	16 (15.1%)	0.020
No	14 ( 63.6% )	90 (84.9%)	
Blisters			
Yes	2 (66.7%)	22 (17.6%)	0.031
No	1 (33.3%)	103 (82.4%)	

Knowledge about foot care

Our study (Table 1) reported that about 72% of the population was unaware of proper foot care practices; in addition to that, only 24 (19%) out of 128 use proper footwear, such as microcellulose rubber (MCR).

Risk classification based on the comprehensive foot examination by Boulton AJ et al. [9]

About 36% of the patients were combinedly qualified for the foot at risk under categories 1, 2, and 3, among which six (5%) were placed under category 1. Eighteen (14%) patients were classified under risk category 2 with LOPS + PAD and 22 (17%) under category 3 with a history of ulcer and/or amputation (Table 4).

**Table 4:** Risk classification based on the comprehensive foot examination LOPS: loss of protective sensation, PAD: peripheral arterial disease

Risk category of the foot	Description	Number (N = 128)%
0	No LOPS, no PAD, no deformity	82 (64.06%)
1	LOPS + deformity	6 (4.687%)
2	PAD + LOPS	18 (14.060%)
3	History of ulcer + amputation	22 (17.18%)

The following factors such as the duration of diabetes, ingrown toenails, previous foot ulcer, deformity, absent pedal pulses, active ulcers, and blisters were significantly associated with neuropathy detected by a 10 g monofilament (p < 0.05). However, the multivariate analysis between the dependent and independent risk factors for the foot at risk was not statistically significant (Table 5).

**Table 5:** Multivariate logistic regression analysis output of different dependent factors of the study. B: estimated coefficient for the independent variable(s); S.E.: standard error of the estimate; df: degrees of freedom; Sig.: statistical significance;  Exp(B):  odds ratio; C.I.: confidence interval; BMI: body mass index; HBA1C: glycosylated hemoglobin; DM: diabetes mellitus

Variable	B	S.E.	Wald	df	Sig.	Exp(B)	95% C.I. for EXP(B)
Age	0.038	0.055	0.487	1	0.485	1.039	0.933-1.158
Sex	-0.834	1.24	0.453	1	0.501	0.434	0.038-4.936
BMI (kg/m^2^)	0.044	0.126	0.122	1	0.727	1.045	0.816-1.339
HbA1C	0.144	0.188	0.591	1	0.442	1.155	0.800-1.669
DM in years	0.059	0.063	0.877	1	0.349	1.061	0.937-1.201
Previous ulcer	-23.295	11810.01	0	1	0.998	0	-
Previous amputation	2.638	68271.95	0	1	1	13.988	-
Absent pedal pulses	-1.994	1.194	2.787	1	0.095	0.136	0.013-1.415
Active ulcer	-23.028	10619.79	0	1	0.998	0	-
Ingrown toenail	-1.614	1.079	2.235	1	0.135	0.199	0.024-1.652
Calluses	-0.331	1.321	0.063	1	0.802	0.718	0.054-9.563
Blisters	19.384	55186.77	0	1	1	262083987.055	-
Constant	19.339	40192.98	0	1	1	250479834.697	-

## Discussion

The present study revealed that 46 (36%) out of 128 patients had at least one risk factor for the diabetic foot by using the SSDFST. A similar result was reported in Nigeria by Adejumo PO et al. [7], but the result was higher from a center in north India by Jayaprakash P et al. [10]. The difference in the prevalence of the foot at risk in the various studies was due to different criteria or tools used to determine the foot at risk.

Ten gram SWM is one of the most effective, easily available, cheap, and reproducible instruments for the screening and identification of peripheral neuropathy, which has been confirmed in the Seattle diabetes foot study by Boyko EJ et al. [8]. Detecting the risk factors of the diabetic foot at the outset and implementing the strategy to prevent it are the keys to avoiding foot ulcers. The loss of pressure sensation for subsequent ulceration picked up by 10 g SWM has a high predictive value reported in the literature by Mayfield JA et al. [11]. The prevalence of neuropathy in our study was 19%, while a multicentric study from India by Viswanathan V et al. reported that the prevalence of neuropathy was 15% [12]. However, in two separate single-center studies from north India by Kishore S et al., the prevalence varies from 35% [10] to 45% [13]. The difference in the variable prevalence rates is multifactorial and depends upon the criteria used to define neuropathy, the duration of diabetes, and its degree of glycemic control, among others.

Multiple risk components play an amassed role in the development of ulceration in diabetes. The prevalence of ingrown toenails, calluses, fissures, blisters, and deformity was 31%, 17%, 21%, 2%, and 3% in our study using the SSDFST; a similar study done in Guyana by Adeyemi TM et al. reported a prevalence of 20%, 20%, 13%, 4%, and 9%, respectively [14]. All these abnormal foot findings were related to an increased risk of foot ulcers when present in combination rather than individually.

Only 24 (19%) of our study population reported appropriate foot care practices. In a study in north India by Kishore S et al., this number is 20% [13], but western data by Adeyemi TM et al. was higher [14]. This may be due to multiple factors such as lack of awareness, knowledge, trained healthcare professionals, and/or failure of our healthcare system to educate patients regarding the importance of proper preventive foot care. The better foot care practice in the western region may be due to dedicated podiatric care.

In our study, the loss of pedal pulse sensation was found in 18 (14%) of the cases; it was higher in a large population study done in Guyana by Woodbury MG et al. [15] and lower in a study done in Nigeria (1.3%) and north India (8-10%), where all the pulsation was checked manually by Adejumo PO et al. [7]. Asymptomatic peripheral artery disease prevalence ranges from 20% to 50% mentioned in the literature by Tummala S et al. [16]; our patients were asymptomatic without having complaints of intermittent claudications or rest pain, constituting about 14%. 

Patients with multiple risk factors such as diabetic peripheral neuropathy, motor deformity of the diabetic foot, and a history of ulcer or amputation are at greater risk of developing further ulcers than neuropathy plus deformity or neuropathy alone [13].

A systemic review depicted that the prevalence of foot ulcers among diabetic patients ranges from 3% to 13% globally [17]. The prevalence of foot ulcers reported in India from a multicentric study was 7.6% [12], and from multiple clinic-based surveys as a part of the Diabetic Foot Education Program (DFEP), it was 4.7% [18]. Our study reported a history of previous foot ulcers (10, 8%) and active ulcers (11, 9%); this high prevalence is due to the referral bias to tertiary care hospitals. The exact etiology of the ulceration was not known but presumed to be multifactorial.

Overall, based on the results of the SSDFST, it was found that approximately one-third of the patients with diabetes presented with a risk of foot ulcers. The ADA, in agreement with the American Association of Clinical Endocrinologists Task Force, recommended that based on the category of the foot at risk, follow-up and treatment should be planned for the prevention of future DFUs and better foot care management.

Risk classification based on the comprehensive foot examination reported that 5% of our study subjects were in category 1 characterized by the LOPS and deformity, with a higher prevalence reported at 33% (category 1) in a north India study by Kishore S et al. [13]. Both LOP and PAD were present in 14% of our subjects (category 2), with the lower prevalence reported in the same north Indian study (10%). Vascular consultation should be sought in categories 2 and 3 for better diabetic foot care. Footwear prescription should be universal for all categories, but custom-made footwear should be advised especially in category 3. 

The limitation of the study is that it is a cross-sectional study and cannot be extrapolated to the general population of people living with T2DM. Because it is a tertiary healthcare referral center, it might overestimate the prevalence we have quoted.

## Conclusions

At least one-third of our patients showed at least one risk factor for diabetic foot based on the SSDFST. Peripheral diabetic neuropathy was detected in about 19% by using the 10 g SWM. The awareness of proper foot care practice was very poor, which constitutes 19% of our study population. Therefore, we recommend that all diabetes patients should have proper awareness of the examination of the foot by themselves to avoid the dreaded complication of DFU and subsequent foot amputation.
